# Transcranial direct current stimulation combined with rehabilitation training for chronic spinal pain: a systematic review and Meta-analysis

**DOI:** 10.3389/fneur.2026.1928977

**Published:** 2026-08-28

**Authors:** Chenna Li, Lei Wu, Juan Chen, Qi Sun, Ping Cao

**Affiliations:** School of Rehabilitation Engineering, China Civil Affairs University, Beijing, China

**Keywords:** chronic spinal pain, disability, neuromodulation, pain intensity, rehabilitation, systematic review and meta-analysis, transcranial direct current stimulation

## Abstract

**Objective:**

To evaluate the incremental clinical effects and safety of active transcranial direct current stimulation (tDCS) added to rehabilitation in adults with chronic spinal pain.

**Methods:**

PubMed, Web of Science Core Collection, CENTRAL, SPORTDiscus via EBSCOhost, and CNKI were searched from inception to 4 July 2026. Randomized trials evaluating active tDCS combined with rehabilitation were eligible. Primary meta-analyses were restricted to active versus sham tDCS delivered with the same or closely matched rehabilitation programme and used immediate post-intervention endpoint data. Hedges’ g was pooled using restricted maximum-likelihood random-effects models with Knapp–Hartung inference. Risk of bias was assessed using RoB 2, and certainty of evidence was evaluated using GRADE.

**Results:**

Ten independent randomized trials involving 372 participants across all study arms were included. Four trials involving 167 participants contributed to the pain-intensity meta-analysis. The pooled effect favoured active tDCS combined with rehabilitation over sham tDCS combined with matched rehabilitation (Hedges’ g = 0.59, 95% CI 0.11–1.06; *p* = 0.030; I^2^ = 7.3%), although certainty was low. Five trials involving 189 participants contributed to the disability meta-analysis, for which the pooled effect was inconclusive and substantially heterogeneous (g = 0.37, 95% CI − 0.51–1.26; *p* = 0.305; I^2^ = 77.3%; very-low-certainty evidence). Quality of life, psychological, motor, postural, and neurophysiological outcomes showed no consistent additional benefit. Six trials reported adverse-event or tolerability data. No serious adverse events were reported, but monitoring was incomplete and one participant withdrew because of contact dermatitis.

**Conclusion:**

Low-certainty evidence suggests that active tDCS added to matched rehabilitation may provide an immediate reduction in pain intensity in adults with chronic spinal pain. Effects on disability and other clinical outcomes remain uncertain, and the available evidence is insufficient to establish safety or exclude uncommon harms. tDCS should therefore remain an investigational adjunct pending adequately powered sham-controlled trials with matched rehabilitation, longer follow-up, and standardized adverse-event monitoring.

**Systematic Review Registration:**

https://www.crd.york.ac.uk/prospero/display_record.php?ID=CRD420261441551, CRD420261441551.

## 1 Introduction

Low back and neck pain are major contributors to disability worldwide and impose substantial individual and societal burdens (1, 2). For the present review, chronic spinal pain was used as a review-level umbrella term encompassing chronic non-specific low back pain, chronic neck pain, cervical or lumbosacral radicular pain, and cervicogenic headache. These conditions share persistent pain and activity limitation, but they are not clinically or mechanistically interchangeable. Pain-related disability is influenced by interacting physical, psychological, and behavioural factors, while nociceptive, neuropathic, and nociplastic mechanisms may occur in different proportions across and within diagnostic groups (3, 4). Radicular pain involves nerve-root-related pathology, whereas cervicogenic headache reflects pain referred from cervical structures through convergence within the trigeminocervical system (5). A broad review-level scope may therefore identify evidence across spinal pain conditions, but diagnosis-specific differences must be considered when interpreting pooled effects.

Exercise therapy and structured rehabilitation are central components of conservative management, particularly for chronic low back pain (6, 7). These interventions can improve pain and function, although their average effects are generally modest and vary across programme types (6). Persistent spinal pain is also not explained solely by local structural or biomechanical abnormalities. Altered descending pain modulation and changes in pain-related and sensorimotor networks have been identified in some individuals with chronic low back pain (8). Neuroplastic adaptations may occur across cortical, spinal, muscular, and behavioural levels, but their expression is heterogeneous and no single central mechanism characterizes all patients (9, 10). These observations provide a rationale for investigating whether centrally directed neuromodulation can augment the effects of task-specific rehabilitation.

Transcranial direct current stimulation is a non-invasive neuromodulation technique that applies low-intensity direct current through scalp electrodes to modify cortical excitability and network activity (11). Motor-cortex and prefrontal stimulation have been investigated in chronic pain because these targets may influence descending pain modulation, sensorimotor processing, and the cognitive–affective dimensions of pain (12, 13). Experimental and pilot clinical evidence nevertheless indicates that target engagement does not necessarily produce uniform changes in pain or function, and responses may depend on the stimulation target, montage, dose, treatment schedule, participant characteristics, and concurrent behavioural task (14). Combining tDCS with rehabilitation or peripheral stimulation has therefore been proposed as a means of pairing central modulation with task-specific sensory and motor input (15, 16). Under this framework, tDCS is evaluated as a potential adjunct to active rehabilitation rather than as a replacement for it.

Previous evidence remains inconsistent, partly because reviews have addressed different clinical and methodological questions. Reviews limited to tDCS or non-invasive brain stimulation for non-specific chronic low back pain have reported limited or uncertain effects on pain and disability (17, 18). By contrast, a meta-analysis of non-invasive brain stimulation combined with exercise and a subsequent review of tDCS combined with peripheral stimulation reported more favourable pain effects across chronic pain populations (19, 20). A broader review of orthopedic pain also included clinically diverse disorders and stimulation contexts (21). A recent meta-analysis of chronic primary pain similarly reported favourable effects of non-invasive brain stimulation on pain intensity, emotional distress, and functional disability, but included multiple stimulation modalities and a broad range of primary pain conditions (22). More recent syntheses have differed further in scope: Xia et al. (23) focused on tDCS combined with exercise for chronic low back pain, Zhou et al. (24) combined standalone and adjunctive tDCS protocols, and Du et al. (25) evaluated several non-invasive brain-stimulation modalities and comparator types. Such differences in population, stimulation modality, comparator, co-intervention, and outcome selection mean that these reviews estimate related but non-equivalent treatment effects. Consequently, the specific additional effect of active tDCS beyond the same or closely matched rehabilitation programme remains insufficiently resolved.

This systematic review therefore evaluated the effects and safety of active tDCS combined with rehabilitation in adults with chronic spinal pain. The primary question was whether active tDCS provides an additional immediate effect on pain intensity or disability compared with sham tDCS delivered alongside the same or closely matched rehabilitation programme. Secondary patient-reported, functional, mechanistic, and safety outcomes were also synthesized, with interpretation accounting for clinical heterogeneity, risk of bias, and certainty of evidence.

## 2 Methods

### 2.1 Reporting guideline, protocol registration, and deviations

This systematic review and meta-analysis was conducted in accordance with the Cochrane Handbook for Systematic Reviews of Interventions and reported according to PRISMA 2020 (26, 27). The review was registered in PROSPERO on 4 July 2026 (CRD420261441551).

The registered population and intervention scope were retained. During revision, the primary meta-analyses were restricted to active versus sham tDCS delivered with the same or closely matched rehabilitation programme, immediate post-intervention endpoint data, and clinically comparable outcome constructs. Trials with rehabilitation-only comparators or shared electrical co-interventions were retained for narrative synthesis, and change scores were not combined with endpoint data. These refinements were made to improve clinical comparability and interpretability.

### 2.2 Search strategy

PubMed, Web of Science Core Collection, CENTRAL, SPORTDiscus via EBSCOhost, and CNKI were searched from inception to 4 July 2026. Eligibility was limited to reports published in English or Chinese because reliable translation and outcome verification in other languages were not available to the review team. Free-text terms for tDCS and chronic spinal pain were adapted for each database. Rehabilitation-related terms were not required in the electronic searches in order to maximize search sensitivity. Reference lists of included studies and relevant reviews were also screened. Unpublished studies were not sought. Full search strategies, search dates, and record counts are provided in Supplementary Table S1.

### 2.3 Eligibility criteria

Eligibility criteria were defined using the Population, Intervention, Comparator, Outcome, and Study design (PICOS) framework.

#### 2.3.1 Participants

Eligible studies included adults aged 18 years or older with chronic non-specific low back pain, chronic neck pain, cervical radiculopathy, lumbosacral radicular pain, or cervicogenic headache. Studies of mixed pain populations were eligible only when data for participants with chronic spinal pain were separately extractable.

Studies involving children or adolescents, healthy participants, acute or postoperative spinal pain, cancer-related pain, fracture, infection, inflammatory spinal disease, severe neurological disease, or non-spinal pain were excluded.

#### 2.3.2 Interventions

The experimental intervention was active tDCS combined with structured rehabilitation training. Eligible rehabilitation interventions included exercise or physical therapy programmes such as motor-control exercise, sensorimotor training, stabilization exercise, neck stabilization training, and cranio-cervical flexion training.

Studies using transcranial magnetic stimulation, transcranial alternating current stimulation, or another non-tDCS brain-stimulation technique were excluded. Studies were also excluded when an additional active treatment was provided only to the experimental group and the specific effect of tDCS could not be isolated.

#### 2.3.3 Comparators

At the systematic-review level, eligible comparators included sham tDCS combined with the same or a closely matched rehabilitation programme and, when relevant to the review question, rehabilitation alone. Rehabilitation was considered closely matched when treatment content, frequency, duration, and therapist contact were comparable between groups.

For the primary meta-analyses, eligibility was restricted to active tDCS plus rehabilitation versus sham tDCS plus the same or closely matched rehabilitation, without an additional shared electrical-stimulation modality. Park et al. (28), which used rehabilitation alone without sham stimulation, and Sharma et al. (29), in which both groups also received transcutaneous electrical nerve stimulation, were therefore retained for narrative synthesis but excluded from the primary meta-analyses. In the three-arm trial by Dere et al. (30), only the active-tDCS and sham-tDCS arms were included in quantitative synthesis.

#### 2.3.4 Outcomes

The primary outcomes were pain intensity and disability. Secondary patient-reported outcomes included quality of life and psychological outcomes. Other eligible outcomes were clinical motor control, muscle strength or endurance, muscle activation, postural control, neurophysiological measures, and adverse events or tolerability.

#### 2.3.5 Study design

Randomized controlled trials and randomized clinical trials were eligible. Non-randomized studies, observational studies, case reports, case series, reviews, protocols, conference abstracts, animal studies, and reports without an eligible comparator or extractable outcome data were excluded.

### 2.4 Study selection and data extraction

Search records were imported into reference-management software, deduplicated electronically, and manually checked. Two reviewers independently screened titles, abstracts, and full texts. Disagreements were resolved by discussion or consultation with a third reviewer, and full-text exclusion reasons were recorded.

Data were extracted using a piloted form and verified against the full reports. Extracted items included study, participant, intervention, comparator, outcome, analysis, registration, and safety characteristics. Adverse-event reporting was recorded by group where available, and non-reporting was not interpreted as absence of events. Multiple publications from the same trial were linked before synthesis; Jobin et al. (31, 32) were treated as one trial and counted once within each outcome analysis. Detailed data are provided in Supplementary Tables S2–S5.

### 2.5 Outcome selection and criteria for quantitative synthesis

The primary time point was the first assessment immediately after treatment. Follow-up results were summarized separately.

For pain intensity and disability, endpoint means and standard deviations were used. When several eligible measures were reported, the study-designated primary measure was selected. If no primary pain measure was specified, overall or average pain was preferred, followed by pain at rest. For disability, the validated condition-specific measure most relevant to the study population was selected. Selection was not based on effect magnitude or statistical significance.

Change scores were not pooled with endpoint scores, and each trial contributed only one estimate per meta-analysis. Quantitative synthesis required at least three clinically comparable trials using the primary comparator and immediate post-intervention data. Only pain intensity and disability met these criteria; all other outcomes were synthesized narratively.

### 2.6 Risk of bias assessment

Risk of bias was assessed using the Cochrane Risk of Bias 2 tool for the effect of assignment to intervention (33). Assessments were result-specific and were conducted separately for immediate post-intervention pain, disability, and quality-of-life results.

The five domains covered randomization, deviations from intended interventions, missing outcome data, outcome measurement, and selective reporting. Two reviewers independently rated each domain and the overall result as low risk, some concerns, or high risk, with disagreements resolved by consensus or a third reviewer. Detailed signalling-question responses are provided in Supplementary Table S6.

### 2.7 Statistical analysis

Continuous outcomes measured using different instruments were summarized as standardized mean differences using Hedges’ g with 95% confidence intervals (CIs). Effect directions were harmonized so that positive values favoured active tDCS combined with rehabilitation. Effect sizes and sampling variances were calculated using the metafor package in R (34).

Random-effects models were fitted using restricted maximum-likelihood estimation of between-study variance (τ^2^). Because each meta-analysis contained few trials, Knapp–Hartung-adjusted CIs and statistical tests were used for primary inference; conventional normal-approximation results were reported only as supplementary analyses. Heterogeneity was assessed using Cochran’s Q, I^2^, and τ^2^, and 95% prediction intervals were calculated for the primary models.

For studies reporting medians and quartiles, means and standard deviations were estimated using the methods of Luo et al. (35) and Wan et al. (36), respectively. When only the median and interquartile-range width were available, the median was used as an approximation of the mean under an approximate-symmetry assumption. Standard deviations were calculated from standard errors using:

$$\mathrm{SD}=\mathrm{SEM}\times \sqrt{n}\;$$

Sensitivity analyses examined restriction to lumbar-related conditions, exclusion of radicular pain trials, exclusion of converted data when at least three studies remained, and alternative median-to-mean conversions. Leave-one-out analyses were conducted as exploratory influence analyses. Formal subgroup analyses and meta-regression were not performed because of the small number of trials. Funnel plots, Egger’s tests, and trim-and-fill analyses were not conducted because each meta-analysis included fewer than 10 independent trials.

All tests were two-sided, with *p* < 0.05 considered statistically significant. Analyses were conducted in R version 4.5.2 (R Foundation for Statistical Computing, Vienna, Austria) using the meta for package.

### 2.8 Certainty of evidence

Certainty of evidence was assessed using GRADE (37). Randomized evidence started at high certainty and was downgraded, where appropriate, for risk of bias, inconsistency, indirectness, imprecision, or publication bias. Judgments considered effect consistency, heterogeneity, clinical comparability, confidence intervals, sample size, and prediction intervals where available. Publication bias was not assessable because each outcome included fewer than 10 trials. Certainty was rated as high, moderate, low, or very low.

## 3 Results

### 3.1 Study selection

**Figure 1:**
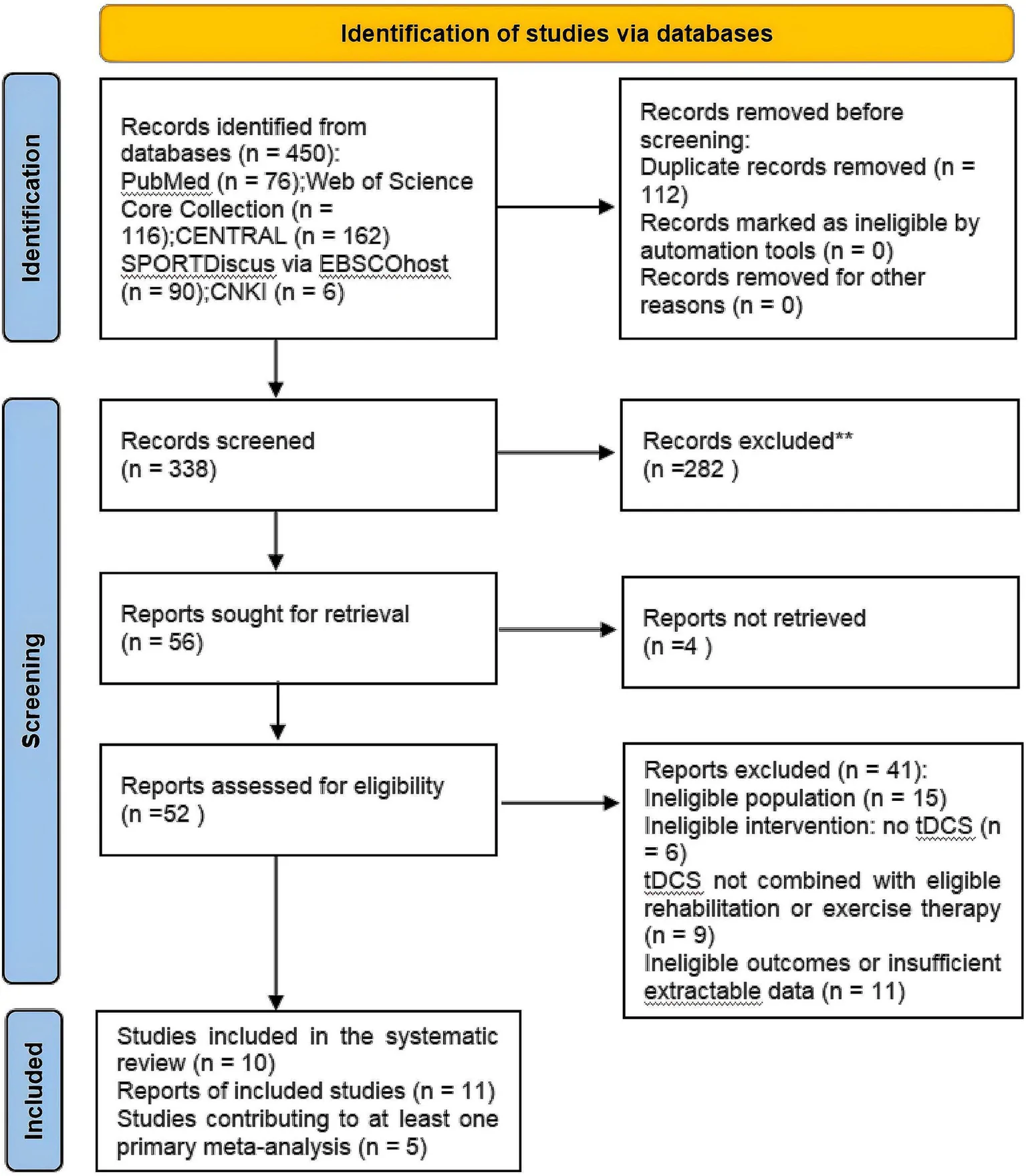
PRISMA 2020 flow diagram.

The database searches identified 450 records: 76 from PubMed, 116 from Web of Science Core Collection, 162 from CENTRAL, 90 from SPORTDiscus via EBSCOhost, and 6 from CNKI. After duplicate removal, title and abstract screening, and full-text assessment, 10 independent randomized trials were included in the systematic review. The two publications by Jobin et al. (31, 32) were treated as reports from one trial to avoid duplicate counting. The study-selection process and aggregate reasons for full-text exclusion are shown in Figure 1.

Four trials contributed to the primary pain-intensity meta-analysis and five to the primary disability meta-analysis. In total, five unique trials contributed to at least one primary meta-analysis, whereas the remaining five trials contributed only to the structured narrative synthesis of secondary, mechanistic, or safety outcomes.

### 3.2 Characteristics of included trials

Although the databases were searched from inception to 4 July 2026, the 10 eligible trials were published between 2018 and 2025. These trials randomized 372 participants across all study arms, with sample sizes ranging from 22 to 74 participants.

Four trials (40%) included participants with chronic non-specific low back pain (38–41), one (10%) included chronic neck pain (30), one (10%) included cervical radiculopathy (29), two (20%) included lumbosacral radicular pain (42, 43), and two (20%) included cervicogenic headache (28, 31, 32).

Nine trials used a sham-tDCS comparator. Park et al. (28) compared tDCS plus cranio-cervical flexion training with training alone, whereas Sharma et al. (29) compared active and sham tDCS in the context of shared transcutaneous electrical nerve stimulation and neck-specific exercise. Dere et al. (30) included active-tDCS, sham-tDCS, and exercise-only groups; only the active and sham groups were included in the primary quantitative synthesis.

Rehabilitation programmes included general exercise, motor-control exercise, sensorimotor training, stabilization exercise, neck-specific exercise, and cranio-cervical flexion training. Stimulation targets, doses, treatment schedules, rehabilitation protocols, and outcome measures varied across trials. Pain was assessed using visual analogue or numeric rating scales, whereas disability was assessed using condition-specific instruments. Detailed study characteristics and trial-report linkage are presented in Table 1 and Supplementary Table S2.

**Table 1:** Characteristics of the included randomized trials.

Study (country)	Condition	Sample, active/control	Age, years, active/control	Sex, male/female, active/control	Active intervention	Comparator	Treatment schedule	Outcomes assessed
Straudi et al. (41) Italy	Chronic non-specific low back pain	18/17	54.3 ± 12.4/56.0 ± 12.9	6/12 / 3/14	Real tDCS plus group exercise	Sham tDCS plus group exercise	5 tDCS sessions followed by 10 group-exercise sessions	VAS, RMDQ, EQ-5D, PHQ-9, adverse events
Leng Leng (38) China	Chronic non-specific low back pain	37/37	40.48 ± 11.47/40.17 ± 12.08	14/23 / 12/25	Active tDCS plus group exercise therapy	Sham tDCS plus group exercise therapy	5 days of tDCS; group exercise therapy for two treatment courses	VAS, RMDQ, EQ-5D, PHQ-9, adverse events
Lu et al(39) China	Chronic non-specific low back pain	20/20	37.75 ± 8.19/34.95 ± 8.10	5/15 / 8/12	Bilateral M1 anodal tDCS plus exercise therapy	Sham tDCS plus the same exercise therapy	4 weeks; 3 sessions/week	NRS, ODI, PCS, SAS, SDS
Sharma et al. (29) India	Cervical radiculopathy	22/22	39.11 (95% CI 30.05–48.18) / 41.89 (95% CI 36.12–47.66)	12/10 / 14/8	Active anodal tDCS plus TENS and neck-specific exercises	Sham tDCS plus TENS and the same neck-specific exercises	4 weeks; 5 sessions/week	NPRS, NDI
Qanbari et al. (43) Iran	Lumbosacral radiculopathy	17/17	35.71 ± 8.53/42.53 ± 11.41	11/6 / 7/10	Real tDCS plus sensory-motor exercises	Sham tDCS plus sensory-motor exercises	Post-test assessment within 24–48 h after intervention	Pain intensity, disability, motor control test, MEP, AMT, N80/N150
Sornkaew et al. (40) Thailand	Chronic low back pain with movement-control impairment	12/10	29.2 ± 7.0/29.8 ± 8.0	5/7 / 5/5	Anodal tDCS plus motor-control exercise	Sham tDCS plus the same motor-control exercise	6 weeks; 12 sessions	MODQ, SF-36, movement-control-impairment test battery, lumbar multifidus activation, cortical topography
Park et al. (28) South Korea	Cervicogenic headache	15/15	31.0 ± 6.5/32.0 ± 5.0	4/11 / 5/10	tDCS plus cranio-cervical flexion exercise	Cranio-cervical flexion exercise alone	4 weeks	NDI, sternocleidomastoid muscle activity
Jobin et al. (31, 32) Canada	Cervicogenic headache	16/16	46.0 (37.8, 56.3) / 52.5 (42.5, 61.5)	6/10 / 7/9	Active tDCS plus exercise therapy	Sham tDCS plus exercise therapy	6 weeks; 18 tDCS sessions plus daily exercise therapy	Headache pain, neck pain, quality of life, mood, adverse events, CCFT, cervical strength, endurance, ROM
Hejazi et al. (42) Iran	Chronic low back pain / chronic unilateral lumbar radiculopathy-related pain	15/15	40.60 ± 7.17/42.07 ± 9.07	5/10 / 6/9	Sensorimotor training plus active tDCS	Sensorimotor training plus sham tDCS	Post-intervention assessment	APA, CPA, NIEMG, muscle-onset latency
Dere et al. (30) Turkey	Chronic neck pain	10/9	31.00 ± 13.74/33.22 ± 16.38	1/9 / 0/9	Active tDCS plus cervical stabilization exercises	Sham tDCS plus cervical stabilization exercises	8 weeks; 16 sessions	VAS, NBQ, ProFitMap-neck, cervical muscle endurance, PSQI, MoCA, adverse events

Values are mean ± SD unless otherwise indicated. Age was reported with 95% confidence intervals in Sharma et al. (29) and as median (interquartile range) in Jobin et al. (31, 32). Within the sex column, values are male/female for the active group followed by male/female for the control group. AMT, active motor threshold; APA, anticipatory postural adjustment; CCFT, cranio-cervical flexion test; CI, confidence interval; CPA, compensatory postural adjustment; EQ-5D, EuroQol-5 Dimension; M1, primary motor cortex; MEP, motor-evoked potential; MoCA, Montreal Cognitive Assessment; MODQ, Modified Oswestry Disability Questionnaire; NBQ, Neck Bournemouth Questionnaire; NDI, Neck Disability Index; NIEMG, normalized integrated electromyography; NPRS, Numeric Pain Rating Scale; NRS, Numeric Rating Scale; ODI, Oswestry Disability Index; PCS, Pain Catastrophizing Scale; PHQ-9, Patient Health Questionnaire-9; PSQI, Pittsburgh Sleep Quality Index; RMDQ, Roland-Morris Disability Questionnaire; ROM, range of motion; SAS, Self-Rating Anxiety Scale; SDS, Self-Rating Depression Scale; SF-36, 36-item Short Form Health Survey; TENS, transcutaneous electrical nerve stimulation; tDCS, transcranial direct current stimulation; VAS, Visual Analogue Scale.

### 3.3 Risk of bias

Risk-of-bias judgements varied across outcomes. For pain intensity, the result from Qanbari et al. (43) was judged at low risk of bias, whereas the results from Leng (38), Lu et al. (39), and Dere et al. (30) raised some concerns. For disability, the results from Qanbari et al. (43) and Sornkaew et al. (40) were judged at low risk of bias, whereas the results from Leng (38), Lu et al. (39), and Dere et al. (30) raised some concerns. For quality of life, the result from Sornkaew et al. (40) was judged at low risk of bias, whereas the results from Leng (38), Straudi et al. (41), and Jobin et al. (31) raised some concerns.

The main concerns were incomplete reporting of allocation concealment, uncertain blinding for participant-reported outcomes, unavailable prespecified analysis plans, and limitations in missing-data handling. No high-risk result contributed to either primary meta-analysis. Summary judgements are presented in Table 2, and detailed supporting assessments are provided in Supplementary Table S6.

**Table 2:** Result-specific risk-of-bias assessment using the Cochrane Risk of Bias 2 (RoB 2) tool.

Outcome	Study	Result assessed	D1	D2	D3	D4	D5	Overall
Pain intensity	Leng, (38)	VAS at T2	Some concerns	Some concerns	Low risk	Some concerns	Some concerns	Some concerns
Pain intensity	Lu et al. (39)	NRS at 4 weeks	Some concerns	Low risk	Low risk	Some concerns	Some concerns	Some concerns
Pain intensity	Qanbari et al. (43)	Pain intensity at post-test	Low risk	Low risk	Low risk	Low risk	Low risk	Low risk
Pain intensity	Dere et al. (30)	VAS-rest at 8 weeks	Low risk	Low risk	Low risk	Some concerns	Some concerns	Some concerns
Disability	Leng (38)	RMDQ at T2	Some concerns	Some concerns	Low risk	Some concerns	Some concerns	Some concerns
Disability	Lu et al. (39)	ODI at 4 weeks	Some concerns	Low risk	Low risk	Some concerns	Some concerns	Some concerns
Disability	Qanbari et al. (43)	Disability level at post-test	Low risk	Low risk	Low risk	Low risk	Low risk	Low risk
Disability	Sornkaew et al. (40)	MODQ at post-intervention	Low risk	Low risk	Low risk	Low risk	Low risk	Low risk
Disability	Dere et al. (30)	NBQ at 8 weeks	Low risk	Low risk	Low risk	Some concerns	Some concerns	Some concerns
Quality of life	Leng (38)	EQ-5D at T2	Some concerns	Some concerns	Low risk	Some concerns	Some concerns	Some concerns
Quality of life	Straudi et al. (41)	EQ-5D change at T2	Low risk	Low risk	Some concerns	Low risk	Low risk	Some concerns
Quality of life	Sornkaew et al. (40)	SF-36 at post-intervention	Low risk	Low risk	Low risk	Low risk	Low risk	Low risk
Quality of life	Jobin et al. (31)	EQ-5D group-by-time result	Low risk	Low risk	Some concerns	Low risk	Low risk	Some concerns

D1, bias arising from the randomization process; D2, bias due to deviations from intended interventions; D3, bias due to missing outcome data; D4, bias in measurement of the outcome; D5, bias in selection of the reported result.

### 3.4 Meta-analysis

#### 3.4.1 Pain intensity

**Figure 2:**
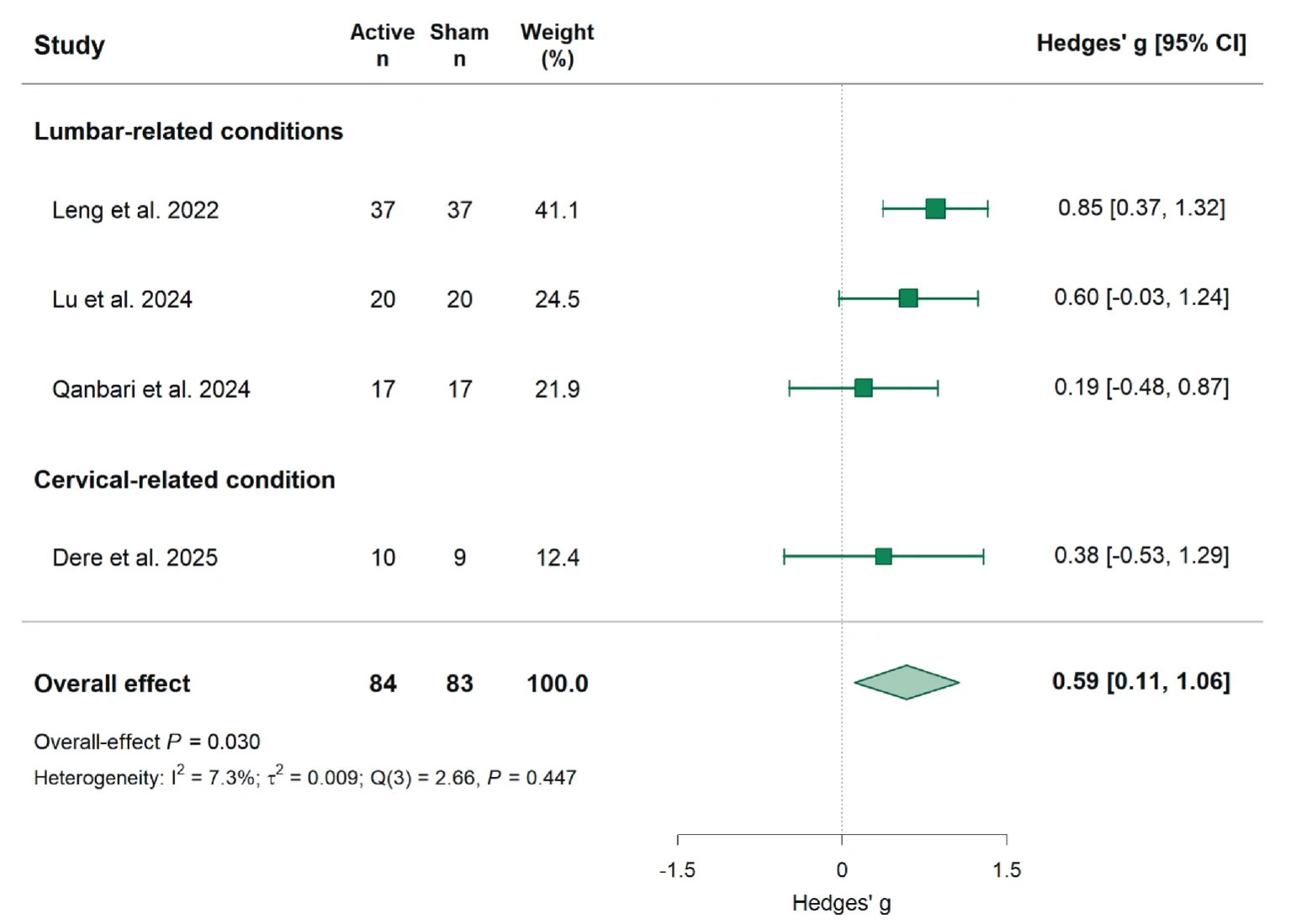
Pain-intensity forest plot (F). Positive Hedges’ *g* values favour active tDCS plus rehabilitation, whereas negative values favour sham tDCS plus rehabilitation.

Four sham-controlled trials involving 167 participants were included. Active tDCS combined with rehabilitation reduced pain intensity compared with sham tDCS combined with matched rehabilitation (Hedges’ g = 0.59, 95% CI 0.11–1.06; *p* = 0.030; I^2^ = 7.3%; τ^2^ = 0.009; 95% PI 0.03–1.15; Figure 2).

The estimate was similar using an alternative data-conversion method (g = 0.60, 95% CI 0.14–1.07). The lumbar-only analysis was inconclusive (g = 0.61, 95% CI − 0.20–1.42), whereas exclusion of the radicular-pain trial retained a favourable effect (g = 0.70, 95% CI 0.17–1.23). Leave-one-out analyses retained favourable point estimates, although several confidence intervals included no effect. Full sensitivity results are provided in Supplementary Table S3.

#### 3.4.2 Disability

**Figure 3:**
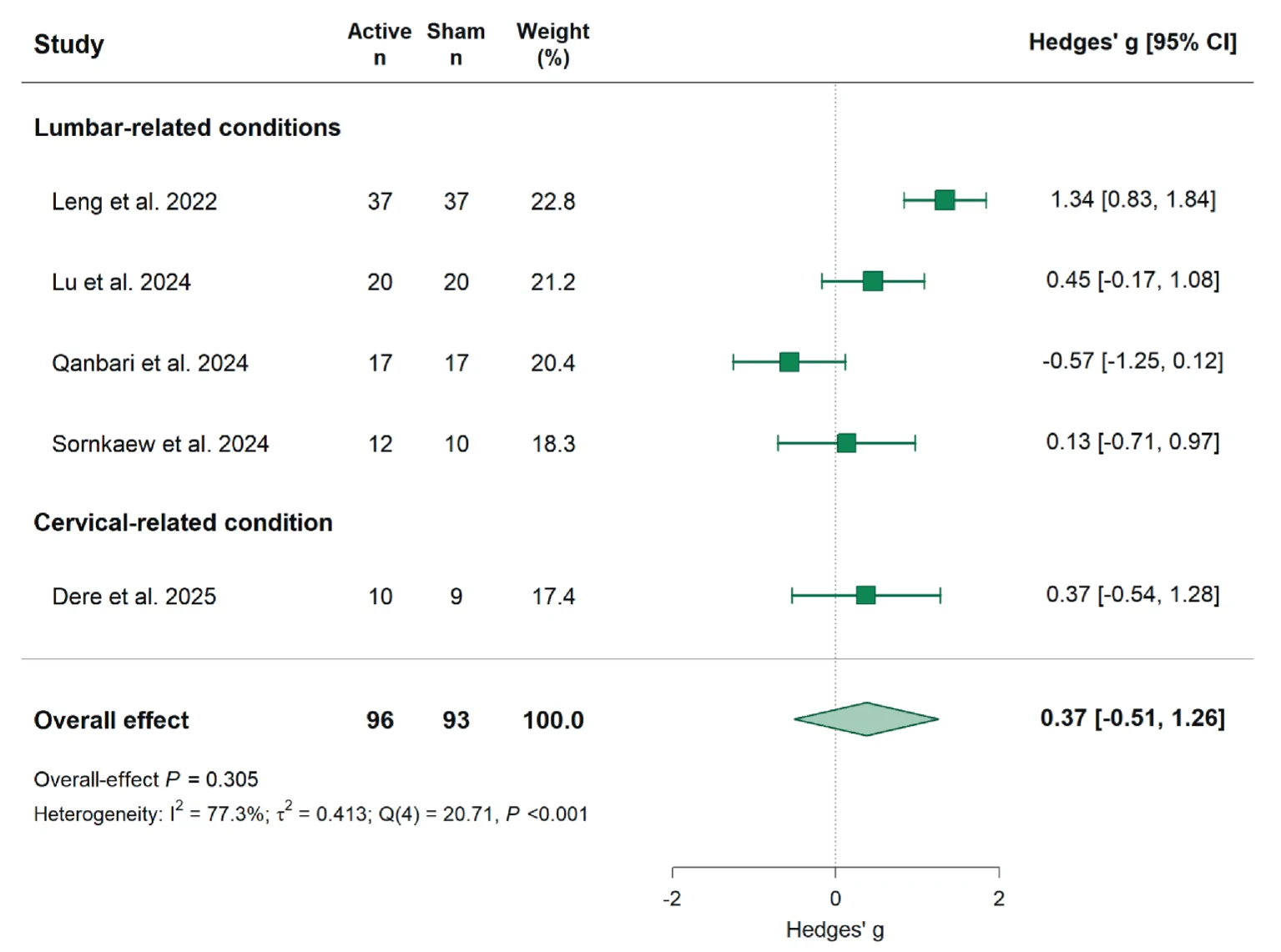
Disability forest plot. Positive Hedges’ *g* values favour active tDCS plus rehabilitation, whereas negative values favour sham tDCS plus rehabilitation.

Five sham-controlled trials involving 189 participants were included. The pooled effect on disability was inconclusive (Hedges’ g = 0.37, 95% CI − 0.51–1.26; *p* = 0.305; I^2^ = 77.3%; τ^2^ = 0.413; 95% PI −1.62–2.36; Figure 3).

The findings remained inconclusive in analyses restricted to lumbar conditions, excluding radicular pain, excluding converted data, using alternative data conversions, and omitting individual trials. Full sensitivity results are provided in Supplementary Table S3.

### 3.5 Secondary outcomes

#### 3.5.1 Quality of life and psychological outcomes

Four trials assessed quality of life. Two reported numerically greater improvements with active tDCS, whereas two found no clear additional benefit on the EQ-5D or SF-36 (31, 38, 40, 41). Overall, the findings were inconsistent and did not demonstrate a clear effect on quality of life.

Four trials assessed depression, anxiety, or pain catastrophizing. Although reductions in PHQ-9 scores favoured active tDCS in two trials, no consistent differences were observed across the PCS, SDS, SAS, GAD-7, or PHQ-9 (31, 38, 39, 41). Psychological outcomes therefore showed no consistent additional benefit.

#### 3.5.2 Motor control and muscle outcomes

Three trials assessed clinical motor control. Active tDCS improved cranio-cervical flexion performance in one trial, whereas no additional effects were found on lumbar motor-control tests in the other two (32, 40, 43).

Muscle-function and activation findings were also mixed. Greater improvement in cervical flexor endurance was reported in one trial, but no consistent effects were observed for cervical extensor or deep-flexor endurance, isometric strength, range of motion, or lumbar multifidus activation (30, 32, 40). Lower sternocleidomastoid activity was reported in a non-sham comparison and was interpreted separately (28). Overall, the evidence did not show a consistent additional effect on motor control or muscle outcomes.

#### 3.5.3 Postural control and neurophysiological outcomes

One trial assessed anticipatory and compensatory postural adjustments. Significant effects were limited to selected muscles and movement phases, whereas no effect was found for muscle-onset latency (42).

Two trials assessed neurophysiological outcomes. Significant changes were reported for selected active motor thresholds, cortical response amplitudes, and cortical-map measures, while most comparisons were not significant (40, 43). These findings were selective and were not consistently accompanied by improvements in clinical outcomes.

Two trials were excluded from the primary meta-analyses because their comparator contexts differed from the primary analysis. Sharma et al. (29) included TENS and exercise in both groups, whereas Park et al. (28) compared tDCS plus exercise with exercise alone; their findings were therefore summarized narratively. Detailed secondary-outcome results are provided in Supplementary Table S4.

### 3.6 Adverse events and tolerability

Six trials involving 226 participants reported adverse-event or tolerability data, whereas four did not describe adverse-event ascertainment or results. No serious adverse events were reported. Minor events were generally transient and included skin redness, tingling or itching, headache, sleepiness, nausea, and dizziness. One participant receiving active tDCS withdrew because of contact dermatitis (31). Given the small samples, short follow-up, and inconsistent monitoring, the evidence was insufficient to establish safety or exclude uncommon harms. Detailed study-level data are provided in Supplementary Table S5.

### 3.7 Certainty of evidence

The GRADE Summary of Findings is presented in Table 3. Certainty was rated low for pain intensity and very low for disability, quality of life, motor control, muscle function and activation, and adverse events. Pain evidence was downgraded for risk of bias and imprecision, whereas disability evidence was downgraded for risk of bias, serious inconsistency, and very serious imprecision. The remaining outcomes were limited by small samples, methodological concerns, inconsistent findings, and imprecision. Publication bias was not assessable because fewer than 10 trials contributed to each outcome.

**Table 3:** GRADE summary of findings for active tDCS plus rehabilitation versus sham tDCS plus matched rehabilitation.

Outcome	Studies (participants)	Effect estimate or synthesis	Certainty of evidence (GRADE)	Interpretation
Pain intensity (Critical)	4 (167)	Hedges’ g = 0.59 SD better (95% CI 0.11 to 1.06 better); I^2^ = 7.3%, τ^2^ = 0.009.	Low ⊕ ⊕◯◯^a^^,^^b^	Active tDCS combined with rehabilitation may reduce pain intensity, but the true effect may range from small to large.
Disability (Critical)	5 (189)	Hedges’ g = 0.37 SD better (95% CI 0.51 SD worse to 1.26 SD better); I^2^ = 77.3%, τ^2^ = 0.413.	Very low ⊕ ◯◯◯^c^^,^^d^	The effect of active tDCS combined with rehabilitation on disability is very uncertain.
Quality of life (Important)	4 (163)	Not pooled. Two studies suggested favorable changes, whereas two showed no clear additional group effect.	Very low ⊕ ◯◯◯^e^	The effect on quality of life is very uncertain, and no consistent additional benefit was demonstrated.
Clinical motor control (Important)	3 (88)	Not pooled. One trial reported improvement in the cranio-cervical flexion test, whereas two found no additional benefit on other motor-control tests.	Very low ⊕ ◯◯◯^f^	Evidence for an additional effect on clinical motor control is very uncertain.
Muscle function and activation (Important)	4 (103)	Not pooled. Isolated improvements occurred for selected cervical endurance or muscle-activity outcomes, without a consistent pattern across studies.	Very low ⊕ ◯◯◯^g^	The effect on muscle function and activation is very uncertain.
Adverse events and tolerability (Critical)	6 (226)	Not pooled. No serious adverse events were reported; one participant withdrew because of contact dermatitis; minor symptoms were generally transient.	Very low ⊕ ◯◯◯^h^	The evidence is insufficient to establish safety or exclude uncommon adverse events.

a Downgraded one level for risk of bias because three of four contributing results had some concerns, mainly owing to incomplete reporting of allocation concealment, blinding integrity, or prospective analysis specification.

b Downgraded one level for imprecision because only four trials (167 participants) were available; the confidence interval ranged from a small to a large benefit, and the lumbar-only sensitivity interval crossed no effect.

c Downgraded one level for risk of bias because three of five contributing results had some concerns.

d Downgraded one level for inconsistency (I^2^ = 77.3%, τ^2^ = 0.413) and two levels for imprecision because the confidence interval included appreciable worsening, no effect, and a large benefit.

e Downgraded for risk of bias, inconsistency, and imprecision. The four trials used different data structures and produced mixed findings, so no pooled estimate was considered clinically defensible.

f Downgraded for inconsistency, indirectness, and imprecision. Three small studies assessed different tests and anatomical regions, and only one reported a clear additional effect.

g Downgraded for risk of bias, inconsistency, indirectness, and imprecision. The evidence combined distinct constructs and included a non-sham comparison.

h Downgraded for limitations in adverse-event ascertainment and reporting, inconsistency in event definitions, and very serious imprecision for uncommon or serious harms.

CI, confidence interval; GRADE, Grading of Recommendations Assessment, Development and Evaluation; SD, standard deviation; tDCS, transcranial direct current stimulation. Publication bias was not formally assessable because fewer than 10 studies contributed to each synthesis.

## 4 Discussion

### 4.1 Principal findings and interpretation

This systematic review included ten independent randomized trials evaluating active tDCS as an adjunct to rehabilitation for chronic spinal pain. Low-certainty evidence suggested a possible immediate reduction in pain intensity when active tDCS was compared with sham stimulation alongside the same or closely matched rehabilitation programme. In contrast, the effect on disability was inconclusive and heterogeneous, while secondary clinical and mechanistic outcomes showed no consistent additional benefit.

No serious adverse events were reported, but incomplete monitoring and small samples precluded firm conclusions about safety.

The pain estimate was consistent in direction across four trials and showed low statistical heterogeneity. Nevertheless, the small evidence base, limited precision, and inconclusive lumbar-only analysis require caution. Moreover, because different pain instruments were expressed as standardized effects, the pooled estimate cannot be translated directly into a single patient-level minimal clinically important difference. The result therefore represents a possible short-term analgesic signal rather than definitive evidence of clinically important pain relief across all chronic spinal pain conditions.

One potential explanation is that tDCS modulates cortical and descending pain-processing systems while rehabilitation provides task-specific sensory and motor input. Clinical studies have suggested that combining central and peripheral stimulation may produce greater analgesic effects than either component alone in some contexts (15, 16). Similar adjunctive effects have been considered in reviews of tDCS combined with physical or peripheral interventions (20, 44). However, differences in stimulation target, montage, dose, treatment timing, rehabilitation content, and participant characteristics prevent identification of the responsible mechanism or optimal protocol.

The absence of a reliable disability effect is not necessarily inconsistent with the pain finding. Disability reflects not only pain intensity but also self-efficacy, fear, psychological distress, physical capacity, and social participation (3). Functional recovery may also require a longer or more behaviorally targeted rehabilitation programme than was provided in the included trials. Exercise and sensorimotor rehabilitation can improve pain and function, but the magnitude and timing of these effects depend on programme content, adherence, and patient characteristics (6, 45). The current evidence therefore does not show that a possible immediate reduction in pain translates into broader functional recovery.

### 4.2 Secondary and mechanistic outcomes

Quality of life and psychological outcomes were measured using different instruments and showed inconsistent findings across small trials. Numerical improvements in quality of life or depressive symptoms were reported in some studies (38, 41), but were not reproduced consistently across the EQ-5D, SF-36, depression, anxiety, and pain-catastrophizing measures (31, 39). Given the very low certainty of evidence, no reliable benefit can be established for these patient-reported outcomes.

Motor control, muscle performance, postural control, and neurophysiological findings were similarly selective. Positive effects were limited to particular clinical tests, muscles, movement phases, or cortical measures, whereas most related comparisons showed no additional effect of active tDCS. For example, improvement in cranio-cervical flexion was not accompanied by consistent changes in other motor or functional outcomes (32). Lumbar motor-control and muscle-activation findings were also largely inconclusive (40, 43). Selected changes in postural adjustments and cortical measures may indicate task-specific target engagement, but they were not consistently associated with improvements in disability or other patient-important outcomes (42). These findings should therefore be regarded as exploratory mechanistic signals rather than evidence of generalized functional recovery.

### 4.3 Clinical heterogeneity and previous evidence

Chronic spinal pain was used as an umbrella term for the review population, but this does not imply clinical or mechanistic equivalence. Nonspecific axial pain, radicular pain, and cervicogenic headache differ in their peripheral drivers, neurological involvement, functional consequences, and rehabilitation priorities. Nociceptive, neuropathic, and nociplastic mechanisms may also coexist in different proportions within the same diagnostic category (4). Cervicogenic headache represents an additional anatomical context because cervical nociceptive input may be referred through the trigeminocervical system (46). Diagnosis-specific responses to tDCS therefore remain plausible.

The primary analyses reduced this heterogeneity by restricting quantitative synthesis to sham-controlled comparisons with the same or closely matched rehabilitation programme and comparable immediate post-intervention outcomes. Diagnosis-restricted analyses were used to assess robustness rather than to test adequately powered subgroup effects. The lumbar-only pain estimate remained favourable in direction but became imprecise and crossed the line of no effect. Consequently, the low statistical heterogeneity in the main pain analysis should not be interpreted as evidence that the included conditions were clinically homogeneous or equally responsive.

Differences in review scope also help explain variation from previous findings. Evidence restricted to nonspecific chronic low back pain has previously been judged uncertain (17). Reviews of tDCS combined with physical therapy or peripheral stimulation have reported more favorable pain effects but included broader co-intervention contexts (20, 44). More recent syntheses have variously examined tDCS combined specifically with exercise, both standalone and combined tDCS protocols, or several non-invasive brain-stimulation modalities (23, 24). The broader analysis by Du et al. (25) also included multiple stimulation techniques and comparator types. These reviews therefore estimate related but non-equivalent treatment effects. The present review provides a narrower estimate of the additional effect of active tDCS beyond matched rehabilitation.

### 4.4 Safety and clinical implications

No serious adverse events were reported, and the reported minor symptoms were generally transient. However, only six trials provided usable adverse-event or tolerability data, monitoring procedures varied, and follow-up was usually short. One participant receiving active tDCS withdrew because of contact dermatitis. The findings therefore indicate only that serious harms were not observed in these small trials; they do not establish safety or exclude uncommon, delayed, or protocol-specific adverse events.

The broader tDCS literature suggests that conventional low-intensity stimulation is most commonly associated with transient scalp sensations and mild headache when established procedures are followed (47). Current guidance nevertheless emphasizes systematic screening, appropriate dosing, trained supervision, and standardized adverse-event monitoring (48, 49). These broader data provide context but cannot compensate for incomplete safety ascertainment in the included trials.

Active tDCS should therefore remain an investigational adjunct rather than a routine component of rehabilitation for chronic spinal pain. The possible immediate pain effect warrants further evaluation, but current evidence does not establish clinically meaningful or sustained improvements in disability and broader rehabilitation outcomes. tDCS should not replace evidence-based rehabilitation and should be evaluated within appropriately monitored research or specialist settings. This interpretation is consistent with recommendations that neuromodulation be incorporated cautiously into multimodal chronic pain care (12).

### 4.5 Strengths, limitations, and research priorities

This review distinguished the broad clinical scope of the systematic review from the stricter criteria used for quantitative synthesis. The primary analyses were limited to active-versus-sham comparisons with matched rehabilitation, used one estimate from each independent trial per outcome, and did not combine endpoint with change-score data. Reports from the same trial were linked to prevent duplicate counting. Result-specific risk-of-bias assessment, GRADE evaluation, and sensitivity analyses further supported outcome-specific interpretation.

The evidence nevertheless remains limited. Only four trials contributed to the pain analysis and five to the disability analysis, resulting in small cumulative samples and limited precision. Diagnoses, stimulation protocols, rehabilitation programmes, and treatment durations differed across studies, restricting generalizability. Some data required conversion from medians and quartiles, although sensitivity analyses did not materially alter the main interpretations. Allocation concealment, prespecified analyses, and blinding procedures were incompletely reported in several trials. The credibility of ramp-up and ramp-down sham stimulation may also decline during repeated or higher-intensity sessions and should be assessed rather than assumed (50, 51). Follow-up was generally short, adverse-event monitoring was inconsistent, and the small number of studies prevented reliable subgroup analysis, meta-regression, or formal assessment of publication bias.

Future trials should compare active and credible sham tDCS against an identical rehabilitation background, with other co-interventions balanced between groups. Studies should be adequately powered and should either focus on clinically coherent diagnostic populations or prespecify stratified analyses. Prospective registration, accessible analysis plans, assessment of blinding credibility, and reporting of between-group estimates with confidence intervals are needed. Pain, disability, quality of life, participant-reported global improvement, and longer-term follow-up should be prioritized. Motor and neurophysiological outcomes should be treated as mechanistic unless their relationship with patient-important benefit is demonstrated, and standardized adverse-event monitoring should be incorporated into every trial.

## 5 Conclusion

Low-certainty evidence suggests that active tDCS added to the same or closely matched rehabilitation programme may provide an immediate reduction in pain intensity in adults with chronic spinal pain. Effects on disability and other clinical or mechanistic outcomes remain uncertain, and incomplete adverse-event reporting prevents firm conclusions about safety. tDCS should therefore be considered an investigational adjunct rather than a routine component of rehabilitation. Adequately powered sham-controlled trials with matched rehabilitation, longer follow-up, and standardized safety monitoring are needed to determine whether any analgesic benefit is reproducible, clinically meaningful, and sustained.

## Data Availability

The raw data supporting the conclusions of this article will be made available by the authors, without undue reservation.
