# Supplementary material for: Transcranial direct current stimulation combined with rehabilitation training for chronic spinal pain: a systematic review and Meta-analysis
Source: Front Neurol. 2026 Aug 28;17:1928977. doi: 10.3389/fneur.2026.1928977 (PMC13561904; doi:10.3389/fneur.2026.1928977)
Supplement: Supplementary file 1 [file Table_1.DOCX]

**Supplementary Table S1. search strategies and record counts**

All databases were searched from inception to 4 July 2026. Searches combined terms for transcranial direct current stimulation with terms for chronic spinal pain. No publication-date restriction was applied. Records shown below are the numbers retrieved before deduplication.

**Overview of database yields**

| **Database** | **Platform/interface** | **Search date** | **Records** |
| --- | --- | --- | --- |
| PubMed | National Library of Medicine | 4 July 2026 | 76 |
| Web of Science Core Collection | Clarivate Web of Science | 4 July 2026 | 116 |
| CENTRAL | Cochrane Library | 4 July 2026 | 162 |
| EBSCOhost | All databases available in the institutional interface (9) | 4 July 2026 | 90 |
| CNKI | China National Knowledge Infrastructure | 4 July 2026 | 6 |
| **Total** |  |  | **450** |

**PubMed** *(National Library of Medicine; searched 4 July 2026)*

| **Line** | **Search strategy** | **Records** |
| --- | --- | --- |
| #1 | ("transcranial direct current stimulation"[Title/Abstract] OR "transcranial direct-current stimulation"[Title/Abstract] OR "transcranial DC stimulation"[Title/Abstract] OR tDCS[Title/Abstract] OR "HD-tDCS"[Title/Abstract] OR "high-definition transcranial direct current stimulation"[Title/Abstract]) | — |
| #2 | ("low back pain"[Title/Abstract] OR "chronic low back pain"[Title/Abstract] OR "nonspecific low back pain"[Title/Abstract] OR "non-specific low back pain"[Title/Abstract] OR "back pain"[Title/Abstract] OR "neck pain"[Title/Abstract] OR "chronic neck pain"[Title/Abstract] OR cervicalgia[Title/Abstract] OR "cervicogenic headache"[Title/Abstract] OR "cervicogenic pain"[Title/Abstract] OR "spinal pain"[Title/Abstract] OR "lumbosacral radiculopathy"[Title/Abstract] OR "lumbar radiculopathy"[Title/Abstract] OR "cervical radiculopathy"[Title/Abstract] OR "radicular pain"[Title/Abstract]) | — |
| #3 | #1 AND #2 | **76** |

*Records retrieved: 76.*

**Web of Science Core Collection** *(Clarivate; searched 4 July 2026)*

| **Line** | **Search strategy** | **Records** |
| --- | --- | --- |
| #1 | TS=("transcranial direct current stimulation" OR "transcranial direct-current stimulation" OR "transcranial DC stimulation" OR tDCS OR "HD-tDCS" OR "high-definition transcranial direct current stimulation") | — |
| #2 | TS=("low back pain" OR "chronic low back pain" OR "nonspecific low back pain" OR "non-specific low back pain" OR "back pain" OR "neck pain" OR "chronic neck pain" OR cervicalgia OR "cervicogenic headache" OR "cervicogenic pain" OR "spinal pain" OR "lumbosacral radiculopathy" OR "lumbar radiculopathy" OR "cervical radiculopathy" OR "radicular pain") | — |
| #3 | #1 AND #2 | **116** |

*Records retrieved: 116.*

**Cochrane Central Register of Controlled Trials (CENTRAL)** *(Cochrane Library; searched 4 July 2026)*

*All databases were searched from inception to 4 July 2026. The electronic searches combined free-text terms for transcranial direct current stimulation with terms for chronic spinal pain. Rehabilitation-related terms were not required in order to maximize sensitivity. No publication-date restriction was applied. Records shown below are the numbers retrieved before deduplication.*

| **Line** | **Search strategy** | **Records** |
| --- | --- | --- |
| #1 | ("transcranial direct current stimulation" OR "transcranial direct-current stimulation" OR "transcranial DC stimulation" OR tDCS OR "HD-tDCS" OR "high-definition transcranial direct current stimulation"):ti,ab,kw | — |
| #2 | ("low back pain" OR "chronic low back pain" OR "nonspecific low back pain" OR "non-specific low back pain" OR "back pain" OR "neck pain" OR "chronic neck pain" OR cervicalgia OR "cervicogenic headache" OR "cervicogenic pain" OR "spinal pain" OR "lumbosacral radiculopathy" OR "lumbar radiculopathy" OR "cervical radiculopathy" OR "radicular pain"):ti,ab,kw | — |
| #3 | #1 AND #2 | **162** |

*Records retrieved: 162.*

**EBSCOhost** *(SPORTDiscus via EBSCOhost in the institutional interface (9); searched 4 July 2026)*

| **Line** | **Search strategy** | **Records** |
| --- | --- | --- |
| #1 | (TI ("transcranial direct current stimulation" OR "transcranial direct-current stimulation" OR "transcranial DC stimulation" OR tDCS OR "HD-tDCS" OR "high-definition transcranial direct current stimulation") OR AB ("transcranial direct current stimulation" OR "transcranial direct-current stimulation" OR "transcranial DC stimulation" OR tDCS OR "HD-tDCS" OR "high-definition transcranial direct current stimulation")) | — |
| #2 | (TI ("low back pain" OR "chronic low back pain" OR "nonspecific low back pain" OR "non-specific low back pain" OR "back pain" OR "neck pain" OR "chronic neck pain" OR cervicalgia OR "cervicogenic headache" OR "cervicogenic pain" OR "spinal pain" OR "lumbosacral radiculopathy" OR "lumbar radiculopathy" OR "cervical radiculopathy" OR "radicular pain") OR AB ("low back pain" OR "chronic low back pain" OR "nonspecific low back pain" OR "non-specific low back pain" OR "back pain" OR "neck pain" OR "chronic neck pain" OR cervicalgia OR "cervicogenic headache" OR "cervicogenic pain" OR "spinal pain" OR "lumbosacral radiculopathy" OR "lumbar radiculopathy" OR "cervical radiculopathy" OR "radicular pain")) | — |
| #3 | #1 AND #2 | **90** |

*Records retrieved: 90.*

**China National Knowledge Infrastructure (CNKI)** *(CNKI; searched 4 July 2026)*

| **Line** | **Search strategy** | **Records** |
| --- | --- | --- |
| #1 | 主题 = 经颅直流电 | — |
| #2 | 主题 = 腰痛 | — |
| #3 | #1 AND #2 | **6** |

*Records retrieved: 6.*

**Abbreviations:** AB, abstract; CNKI, China National Knowledge Infrastructure; CENTRAL, Cochrane Central Register of Controlled Trials; HD-tDCS, high-definition transcranial direct current stimulation; TI, title; TS, topic; tDCS, transcranial direct current stimulation.

*Note: The retained EBSCOhost screenshot identified the search source as “All databases (9)”; the individual database names were not displayed in the screenshot.*
