# Supplementary material for: Transcranial direct current stimulation combined with rehabilitation training for chronic spinal pain: a systematic review and Meta-analysis
Source: Front Neurol. 2026 Aug 28;17:1928977. doi: 10.3389/fneur.2026.1928977 (PMC13561904; doi:10.3389/fneur.2026.1928977)
Supplement: Supplementary file 2 [file Table_2.DOCX]

**Supplementary Table S2. Trial-report linkage and synthesis role of the included randomized trials**

*The unit of counting is the independent randomized trial. Multiple publications from the same trial are linked and counted once.*

| **Trial ID** | **Report(s)** | **Condition** | **Active/control n** | **Active intervention** | **Comparator** | **Sham controlled** | **Shared additional intervention** | **Primary quantitative-synthesis role** | **Narrative role and linkage note** |
| --- | --- | --- | --- | --- | --- | --- | --- | --- | --- |
| Straudi 2018 | Straudi et al. 2018 | Non-specific chronic low back pain | 18/17 | real tDCS + group exercise | sham tDCS + group exercise | Yes | None | Excluded from the primary endpoint meta-analysis because only change-score data were available; retained for narrative synthesis. | QoL, psychological and safety narrative/sensitivity. One report / one trial |
| Leng 2022 | Leng Bo 2022 | Non-specific chronic low back pain | 37/37 | tDCS + group exercise | sham tDCS + group exercise | Yes | None | Eligible by outcome/data type | QoL, psychological, safety as applicable. One report / one trial |
| Lu 2024 | Lu Xuefang et al. 2024 | Non-specific chronic low back pain | 20/20 | bilateral M1 anodal tDCS + exercise | sham tDCS + exercise | Yes | None | Eligible after documented data conversion | Psychological outcomes narrative. One report / one trial |
| Sharma 2025 | Sharma et al. 2025 | Cervical radiculopathy | 22/22 | active tDCS + TENS + neck-specific exercise | sham tDCS + TENS + same exercise | Yes | TENS in both groups | Exclude from primary meta | Narrative evidence in a TENS background. One report / one trial; final citation year 2025 |
| Qanbari 2024 | Qanbari et al. 2024 | Lumbosacral radiculopathy | 17/17 | real tDCS + sensory-motor exercise | sham tDCS + sensory-motor exercise | Yes | None | Eligible for pain/disability | Motor control and neurophysiology narrative. Independent trial |
| Sornkaew 2024 | Sornkaew et al. 2024 | Chronic low back pain with movement-control impairment | 12/10 | anodal tDCS + motor-control exercise | sham tDCS + same exercise | Yes | None | Eligible for disability | QoL, motor control, muscle activation and cortical topography narrative. Independent trial |
| Park 2019 | Park et al. 2019 | Cervicogenic headache | 15/15 | tDCS + cranio-cervical flexion | cranio-cervical flexion only | No | None | Exclude from primary meta | Narrative non-sham comparison. Independent trial |
| Jobin 2025 | Jobin et al. 2025a (Headache); Jobin et al. 2025b (NeuroRehabilitation) | Cervicogenic headache | 16/16 | active tDCS + exercise therapy | sham tDCS + exercise therapy | Yes | None | Not included in quantitative synthesis because comparable group-level endpoint means and standard deviations were unavailable. | Motor control, strength, endurance and ROM narrative; Pain, QoL, mood and safety narrative unless extractable. Two publications from one randomized trial; counted once in the review and never duplicated within an outcome. |
| Hejazi 2025 | Hejazi et al. 2025 | Chronic unilateral lumbar radiculopathy / low back pain | 15/15 | sensorimotor training + tDCS | sensorimotor training + sham tDCS | Yes | None | Narrative postural-control dataset; no clinical pain/disability outcomes | Postural-control mechanisms narrative. Independent registry/trial from Qanbari |
| Dere 2025 | Dere et al. 2025 | Chronic neck pain | 10/9 | active tDCS + stabilization exercise | sham tDCS + stabilization exercise | Yes | None | Eligible using active vs sham arms only | Muscle endurance and other outcomes narrative. Exercise-only arm not used in primary comparison |

Notes: Jobin et al. 2025a and Jobin et al. 2025b are two reports from one trial and are counted as one included study. For Dere et al. 2025, only the active-tDCS and sham-tDCS arms were used in quantitative synthesis. The five unique trials contributing to at least one primary meta-analysis were Leng 2022, Lu 2024, Qanbari 2024, Sornkaew 2024, and Dere 2025.

Abbreviations: M1, primary motor cortex; RCT, randomized controlled trial; TENS, transcutaneous electrical nerve stimulation; tDCS, transcranial direct current stimulation.
