# Supplementary material for: Transcranial direct current stimulation combined with rehabilitation training for chronic spinal pain: a systematic review and Meta-analysis
Source: Front Neurol. 2026 Aug 28;17:1928977. doi: 10.3389/fneur.2026.1928977 (PMC13561904; doi:10.3389/fneur.2026.1928977)
Supplement: Supplementary file 3 [file Table_3.DOCX]

**Supplementary Table S3. Outcome-level data, conversion methods, and quantitative-synthesis decisions for pain intensity and disability**

**Table S3A. Study-level data included in the primary pain-intensity meta-analysis**

| **Study** | **Condition** | **Outcome/time point** | **Active n; mean ± SD** | **Control n; mean ± SD** | **Data type** | **Hedges' g [95% CI]** | **Random-effects weight** | **Decision note** |
| --- | --- | --- | --- | --- | --- | --- | --- | --- |
| Leng 2022 | Non-specific chronic low back pain | VAS; Post exercise T2 | 37; 2.45 ± 0.86 | 37; 3.24 ± 0.98 | endpoint mean±SD | 0.85 [0.37, 1.32] | 41.1% | Standard sham-controlled comparison |
| Lu 2024 | Non-specific chronic low back pain | NRS; Post 4 weeks | 20; 1.23 ± 1.40 | 20; 2.09 ± 1.40 | Luo mean + Wan SD | 0.60 [-0.03, 1.24] | 24.5% | Primary conversion; alternative conversion and exclusion sensitivity prespecified. |
| Dere 2025 | Chronic neck pain | VAS-rest; Post 8 weeks | 10; 0.65 ± 1.22 | 9; 1.30 ± 2.01 | Median as mean + Wan SD | 0.38 [-0.53, 1.29] | 12.4% | Only median and IQR width were reported; exclusion sensitivity prespecified. |
| Qanbari 2024 | Lumbosacral radiculopathy | Pain intensity 0–10; Post-test | 17; 2.53 ± 1.77 | 17; 2.87 ± 1.64 | endpoint mean±SD | 0.19 [-0.48, 0.87] | 21.9% | Clinical interaction nonsignificant |

**Table S3B. Study-level data included in the primary disability meta-analysis**

| **Study** | **Condition** | **Outcome/time point** | **Active n; mean ± SD** | **Control n; mean ± SD** | **Data type** | **Hedges' g [95% CI]** | **Random-effects weight** | **Decision note** |
| --- | --- | --- | --- | --- | --- | --- | --- | --- |
| Leng 2022 | Non-specific chronic low back pain | RMDQ; Post exercise T2 | 37; 3.14 ± 1.50 | 37; 5.43 ± 1.87 | endpoint mean±SD | 1.34 [0.83, 1.84] | 22.8% | Standard sham-controlled comparison |
| Lu 2024 | Non-specific chronic low back pain | ODI; Post 4 weeks | 20; 0.097 ± 0.064 | 20; 0.149 ± 0.144 | Luo mean + Wan SD | 0.45 [-0.17, 1.08] | 21.2% | ODI proportion; alternative conversion and exclusion sensitivity prespecified. |
| Dere 2025 | Chronic neck pain | NBQ; Post 8 weeks | 10; 20.40 ± 13.06 | 9; 24.66 ± 8.13 | endpoint mean±SD | 0.37 [-0.54, 1.28] | 17.4% | Active vs sham arms only |
| Qanbari 2024 | Lumbosacral radiculopathy | Disability level; Post-test | 17; 7.40 ± 4.27 | 17; 5.20 ± 3.23 | endpoint mean±SD | -0.57 [-1.25, 0.12] | 20.4% | Endpoint favors sham; interaction nonsignificant |
| Sornkaew 2024 | Chronic low back pain with movement-control impairment | MODQ; Post-intervention | 12; 4.50 ± 4.80 | 10; 5.30 ± 6.80 | endpoint mean±SD | 0.13 [-0.71, 0.97] | 18.3% | Standard sham-controlled comparison |

**Table S3C. Relevant clinical datasets not included in the primary endpoint meta-analyses**

| **Domain** | **Study** | **Outcome** | **Time point** | **Active data** | **Control data** | **Data structure** | **Reason excluded from primary meta-analysis** | **Narrative use/note** |
| --- | --- | --- | --- | --- | --- | --- | --- | --- |
| Pain | Straudi 2018 | VAS change | T2 change after exercise | 18; -21.20 ± 30.70 | 17; -7.20 ± 32.50 | change mean±SD | Change score not mixed with primary endpoints |  |
| Disability | Straudi 2018 | RMDQ change | T2 change after exercise | 18; -3.80 ± 3.70 | 17; -3.50 ± 4.20 | change mean±SD | Change score not mixed with primary endpoints |  |
| Pain | Sharma 2025 | NPRS | Post 4 weeks | 22; 3.00 ± 0.74 | 22; 4.00 ± 0.37 | median/IQR converted | Common TENS creates distinct treatment context | Retain result narratively |
| Disability | Sharma 2025 | NDI | Post 4 weeks | 22; 22.85 ± 6.99 | 22; 35.33 ± 4.41 | SEM converted to SD | Common TENS creates distinct treatment context | NDI is disability, not a separate QoL outcome |
| Disability | Park 2019 | NDI | Post 4 weeks | 15; 17.90 ± 1.00 | 15; 18.90 ± 1.10 | endpoint mean±SD | No sham tDCS comparator | Retain as non-sham evidence |
| Pain | Jobin 2025 | Headache pain / neck pain | Post-treatment | 16; model estimate only | 16; model estimate only | model beta/CI only | Need group means/SD or valid effect conversion | Do not duplicate trial |
| Disability | Jobin 2025 | HDI / PROMIS-PI | Post-treatment | 16; model estimate only | 16; model estimate only | model beta/CI only | Need usable group data | Do not duplicate trial |

**Table S3D. Primary and prespecified sensitivity analyses**

| **Domain** | **Analysis** | **k** | **Participants** | **Hedges' g [95% KH CI]** | **P** | **I²** | **τ²** | **95% prediction interval** |
| --- | --- | --- | --- | --- | --- | --- | --- | --- |
| Pain | Primary endpoint model | 4 | 167 | 0.59 [0.11, 1.06] | 0.030 | 7.3% | 0.009 | [0.03, 1.15] |
| Disability | Primary endpoint model | 5 | 189 | 0.37 [-0.51, 1.26] | 0.305 | 77.3% | 0.413 | [-1.62, 2.36] |
| Pain | Lumbar-related conditions only | 3 | 148 | 0.61 [-0.20, 1.42] | 0.084 | 18.8% | 0.021 | [-0.41, 1.63] |
| Pain | Exclude lumbosacral radiculopathy | 3 | 133 | 0.70 [0.17, 1.23] | 0.029 | 0.0% | 0.000 | [0.17, 1.23] |
| Pain | Exclude median + IQR-width conversion | 3 | 148 | 0.61 [-0.20, 1.42] | 0.084 | 18.8% | 0.021 | [-0.41, 1.63] |
| Pain | Alternative median-to-mean conversion | 4 | 167 | 0.60 [0.14, 1.07] | 0.026 | 4.1% | 0.005 | [0.09, 1.12] |
| Disability | Lumbar-related conditions only | 4 | 170 | 0.37 [-0.91, 1.65] | 0.429 | 83.6% | 0.554 | [-2.33, 3.06] |
| Disability | Exclude lumbosacral radiculopathy | 4 | 155 | 0.64 [-0.24, 1.52] | 0.104 | 63.2% | 0.213 | [-1.07, 2.35] |
| Disability | Exclude Lu quartile-converted data | 4 | 149 | 0.34 [-0.96, 1.64] | 0.462 | 81.6% | 0.577 | [-2.40, 3.09] |
| Disability | Alternative Lu conversion | 5 | 189 | 0.38 [-0.51, 1.26] | 0.301 | 77.3% | 0.414 | [-1.61, 2.37] |

**Table S3E. Leave-one-out influence analyses**

| **Domain** | **Omitted study** | **k** | **Participants** | **Hedges' g [95% KH CI]** | **P** | **I²** | **τ²** |
| --- | --- | --- | --- | --- | --- | --- | --- |
| Pain | Leng 2022 | 3 | 93 | 0.40 [-0.15, 0.96] | 0.088 | 0.0% | 0.000 |
| Pain | Lu 2024 | 3 | 127 | 0.55 [-0.36, 1.45] | 0.122 | 32.7% | 0.056 |
| Pain | Dere 2025 | 3 | 148 | 0.61 [-0.20, 1.42] | 0.084 | 18.8% | 0.021 |
| Pain | Qanbari 2024 | 3 | 133 | 0.70 [0.17, 1.23] | 0.029 | 0.0% | 0.000 |
| Disability | Leng 2022 | 4 | 115 | 0.08 [-0.69, 0.85] | 0.754 | 43.6% | 0.114 |
| Disability | Lu 2024 | 4 | 149 | 0.34 [-0.96, 1.64] | 0.462 | 81.6% | 0.577 |
| Disability | Dere 2025 | 4 | 170 | 0.37 [-0.91, 1.65] | 0.429 | 83.6% | 0.554 |
| Disability | Qanbari 2024 | 4 | 155 | 0.64 [-0.24, 1.52] | 0.104 | 63.2% | 0.213 |
| Disability | Sornkaew 2024 | 4 | 167 | 0.42 [-0.84, 1.69] | 0.367 | 82.8% | 0.539 |

**Table S3F. Conversion methods used for median and quartile data**

| **Component** | **Primary method** | **Equation/implementation** | **Use in this review** | **Sensitivity method** | **Source** |
| --- | --- | --- | --- | --- | --- |
| Estimated mean when Q1, median and Q3 are reported | Luo et al. (2018), Scenario S2 | w = 0.7 + 0.39/n; mean = w×(Q1+Q3)/2 + (1−w)×median | Lu 2024 pain and disability; Sornkaew 2024 motor-control candidate only | Wan et al. mean = (Q1+median+Q3)/3 | https://doi.org/10.1177/0962280216669183 |
| Estimated SD from Q1 and Q3 | Wan et al. (2014), Scenario C2 | SD = (Q3−Q1) / [2×Φ⁻¹((0.75n−0.125)/(n+0.25))] | Lu 2024 pain and disability; Sornkaew 2024 motor-control candidate | SD = IQR/1.35 | https://doi.org/10.1186/1471-2288-14-135 |
| Median and IQR width reported without Q1 and Q3 | Symmetry-based mean approximation plus Wan SD | Mean = median; SD uses the Wan sample-size-adjusted IQR formula | Dere 2025 VAS-rest only | Mean = median; SD = IQR/1.35 | https://doi.org/10.1186/1471-2288-14-135 |
| Interpretive restriction | Converted values are approximations | Do not infer normality from the conversion; identify every converted dataset. | Primary model plus sensitivity analyses excluding or alternatively converting affected studies | Exclude converted studies where k remains sufficient | https://www.math.hkbu.edu.hk/~tongt/papers/median2mean.html |
