# Supplementary material for: Transcranial direct current stimulation combined with rehabilitation training for chronic spinal pain: a systematic review and Meta-analysis
Source: Front Neurol. 2026 Aug 28;17:1928977. doi: 10.3389/fneur.2026.1928977 (PMC13561904; doi:10.3389/fneur.2026.1928977)
Supplement: Supplementary file 4 [file Table_4.DOCX]

**Supplementary Table S4. Structured narrative synthesis of secondary, functional, mechanistic, and non-primary clinical outcomes**

**Table S4A. Quality-of-life and psychological outcomes**

| **Domain** | **Study** | **Outcome** | **Post-treatment result/estimate** | **Study-level interpretation** | **Reason not pooled** |
| --- | --- | --- | --- | --- | --- |
| Quality of life | Leng 2022 | EQ-5D | Active 0.78 ± 0.12; sham 0.68 ± 0.11 at T2 | Numerically favored active tDCS | Only two comparable endpoint trials; not pooled |
| Quality of life | Sornkaew 2024 | SF-36 | Active 71.6 ± 16.2; sham 70.7 ± 8.1; P = 0.51 | No clear additional effect | Different instrument and small evidence base |
| Quality of life | Straudi 2018 | EQ-5D change | Active +0.16 ± 0.20; sham +0.06 ± 0.20 | Numerically favored active tDCS | Change score; not combined with endpoint data |
| Quality of life | Jobin 2025 | EQ-5D | Group×time β = 0.35 (95% CI −1.37 to 2.06); P = 0.690 | No significant group×time effect | Model estimate only; no comparable group mean/SD dataset |
| Psychological | Leng 2022 | PHQ-9 | Active 6.23 ± 1.88; sham 9.54 ± 2.05 at T2 | Numerically favored active tDCS | Depression differs from anxiety/catastrophizing; insufficient comparable trials |
| Psychological | Lu 2024 | PCS | Active 3.50 ± 7.22; sham 3.50 ± 4.44 | No group difference apparent | Converted quartile data; construct-specific |
| Psychological | Lu 2024 | SDS | Active 33.13 ± 9.03; sham 29.38 ± 9.26 | No favorable pattern for active tDCS | Converted quartile data; construct-specific |
| Psychological | Lu 2024 | SAS | Active 31.25 ± 7.87; sham 31.25 ± 6.72 | No group difference apparent | Converted quartile data; construct-specific |
| Psychological | Straudi 2018 | PHQ-9 change | Active −4.4 ± 4.1; sham −1.7 ± 2.2 | Numerically favored active tDCS | Change score; insufficient comparable trials |
| Psychological | Jobin 2025 | PCS, GAD-7, PHQ-9 | β = 0.60 (P = 0.854); 0.71 (P = 0.568); −0.39 (P = 0.765), respectively | No significant group×time effects | Model estimates only; distinct constructs |

**Table S4B. Clinical motor control, muscle function, and muscle activation**

| **Domain** | **Study** | **Outcome** | **Post-treatment result/estimate** | **Study-level interpretation** | **Reason not pooled** |
| --- | --- | --- | --- | --- | --- |
| Clinical motor control | Qanbari 2024 | Motor Control Test | Active 2.07 ± 0.88; sham 2.20 ± 1.32; group×time P = 0.873 | No additional active-tDCS benefit | Different clinical test and lumbar population |
| Clinical motor control | Sornkaew 2024 | Movement-control-impairment battery | Post-treatment median 1.0 in both groups; P = 1.00 | No additional active-tDCS benefit | Different test and data structure |
| Clinical motor control | Jobin 2025 | Cranio-cervical flexion test | Group×time β = 1.571 (95% CI 0.155 to 2.988); P = 0.030 | Favored active tDCS | Cervical/headache-specific test; one small pilot trial |
| Muscle function | Dere 2025 | Cervical flexor endurance | Active 46.37 ± 18.32; sham 30.39 ± 16.07; between-group change P = 0.030 | Favored active tDCS | One selected endurance measure |
| Muscle function | Dere 2025 | Cervical extensor and deep-flexor endurance | Between-group change P = 0.448 and P = 0.698, respectively | No clear additional benefit | Distinct endurance constructs and mixed data formats |
| Muscle function | Jobin 2025 | CEET, CFET, CIST, cervical ROM | Group×time P = 0.815, 0.340, 0.177, and 0.757, respectively | No significant effects | Multiple cervical performance constructs |
| Muscle activation | Sornkaew 2024 | Lumbar multifidus activation | Active 29.49 ± 10.73; sham 30.22 ± 8.64; P = 0.86 | No additional active-tDCS benefit | Mechanistic EMG outcome |
| Muscle activity | Park 2019 | Sternocleidomastoid activity | Active-combination 36.4 ± 3.9% RVC; exercise-only 39.5 ± 2.5% RVC | Favored combined intervention | No sham tDCS; stimulation-specific effect cannot be isolated |

**Table S4C. Postural-control and neurophysiological outcomes**

| **Domain** | **Study** | **Outcome** | **Adjusted or comparative result** | **Study-level interpretation** | **Reason not pooled** |
| --- | --- | --- | --- | --- | --- |
| Postural control | Hejazi 2025 | Ipsilateral TrA/IO NIEMG during CPA1 | Adjusted difference −0.216 (95% CI −0.375 to −0.056); P = 0.010 | Significant muscle- and phase-specific effect | Multiple muscles, sides, and phases |
| Postural control | Hejazi 2025 | Ipsilateral gluteus medius NIEMG during CPA1 | Adjusted difference −0.227 (95% CI −0.362 to −0.093); P = 0.002 | Significant muscle- and phase-specific effect | Multiple muscles, sides, and phases |
| Postural control | Hejazi 2025 | Ipsilateral gluteus medius NIEMG during CPA2 | Adjusted difference −0.113 (95% CI −0.211 to −0.015); P = 0.025 | Significant muscle- and phase-specific effect | Multiple muscles, sides, and phases |
| Postural control | Hejazi 2025 | Contralateral gluteus medius NIEMG during APA1 | Adjusted difference 0.105 (95% CI 0.010 to 0.201); P = 0.032 | Significant muscle- and phase-specific effect | Multiple muscles, sides, and phases |
| Postural control | Hejazi 2025 | Muscle-onset latency | No comparison reached P < 0.05 | No consistent latency effect | Multiple non-significant comparisons |
| Neurophysiology | Qanbari 2024 | Active motor threshold: multifidus | Group×time P = 0.008; interaction d = 1.03 | Favored active tDCS | Physiological surrogate; not directly interchangeable with clinical outcomes |
| Neurophysiology | Qanbari 2024 | Active motor threshold: TrA/IO | Group×time P = 0.029; interaction d = 0.801 | Favored active tDCS | Physiological surrogate |
| Neurophysiology | Qanbari 2024 | N80 amplitude | Group×time P = 0.046; interaction d = 0.729 | Selective neurophysiological effect | Physiological surrogate |
| Neurophysiology | Qanbari 2024 | MEP amplitudes and N150 amplitude | Group×time P = 0.206, 0.633, and 0.377 | No significant interaction | Different neurophysiological constructs |
| Neurophysiology | Sornkaew 2024 | Center-of-gravity distance | Between-group P = 0.020; effect size = 1.07 | Selective cortical-map difference | Most map measures did not differ |
| Neurophysiology | Sornkaew 2024 | Erector-spinae discrete peaks | Between-group P = 0.030 | Selective cortical-map difference | Most threshold, map-volume, and center-of-gravity measures did not differ |

Notes: Results are presented by clinical construct and were not statistically pooled because instruments, anatomical regions, data structures, and mechanistic levels were not sufficiently comparable. Isolated significant mechanistic findings were interpreted as hypothesis-generating and were not treated as evidence of general clinical efficacy.

Abbreviations: APA, anticipatory postural adjustment; CCFT, cranio-cervical flexion test; CEET, cervical extensor endurance test; CFET, cervical flexor endurance test; CIST, cervical isometric strength test; CPA, compensatory postural adjustment; EMG, electromyography; GAD-7, Generalized Anxiety Disorder-7; MEP, motor-evoked potential; NIEMG, normalized integrated EMG; PCS, Pain Catastrophizing Scale; PHQ-9, Patient Health Questionnaire-9; ROM, range of motion; SAS, Self-Rating Anxiety Scale; SDS, Self-Rating Depression Scale; TrA/IO, transversus abdominis/internal oblique.
