# Supplementary material for: Transcranial direct current stimulation combined with rehabilitation training for chronic spinal pain: a systematic review and Meta-analysis
Source: Front Neurol. 2026 Aug 28;17:1928977. doi: 10.3389/fneur.2026.1928977 (PMC13561904; doi:10.3389/fneur.2026.1928977)
Supplement: Supplementary file 5 [file Table_5.DOCX]

**Supplementary Table S5. Adverse-event and tolerability reporting by randomized trial**

**Table S5A. Study-level adverse-event ascertainment and reporting completeness**

| **Study** | **Active/control n** | **Ascertainment method** | **Serious AEs active/control** | **AE withdrawals active/control** | **Follow-up window** | **Reporting judgement** | **Source** |
| --- | --- | --- | --- | --- | --- | --- | --- |
| Leng 2022 | 37/37 | Adverse reactions observed during tDCS treatment | 0/0 | 0/0 | During intervention | Event-specific counts reported; no serious events described | Table 2 |
| Lu 2024 | 20/20 | Not described | NR | NR | Post-treatment | No adverse-event results reported | Full text |
| Straudi 2018 | 18/17 | tDCS side-effects questionnaire after stimulation sessions | 0/0 | 0/0 | Through 1-month follow-up | Mild side-effect frequencies reported | Table 4 |
| Sharma 2025 | 22/22 | Passive/unclear | 0/0 | 0/0 | Post-treatment only | Paper states absence of adverse effects without an event table | Discussion |
| Dere 2025 | 10/9 | Self-reported side effects | 0/0 | 0/0 | During 8-week intervention | Transient symptoms described by group | Results |
| Qanbari 2024 | 17/17 | Not described | NR | NR | Post-test | No adverse-event results reported | Full text |
| Sornkaew 2024 | 12/10 | Participant-reported mild effects; timing not fully specified | 0/0 | 0/0 | During 6-week intervention | Percentages reported for tingling, burning and itching | Results |
| Park 2019 | 15/15 | Not described | NR | NR | Post-treatment | No adverse-event results reported | Full text |
| Jobin 2025 | 16/16 | Serious/minor AE surveillance plus tDCS safety questionnaire before and after each session | 0/0 | 1/0 | Treatment and 12-week follow-up | Most complete safety reporting; one contact dermatitis withdrawal | Tables 4–5 |
| Hejazi 2025 | 15/15 | Not described | NR | NR | Post-treatment | No adverse-event results reported | Full text |

**Table S5B. Event-level adverse-event data reported by the included trials**

| **Study** | **Event** | **Active n/N** | **Control n/N** | **Severity** | **Count status** | **Withdrawal** | **Source** | **Note** |
| --- | --- | --- | --- | --- | --- | --- | --- | --- |
| Leng 2022 | Skin redness | 22/37 | 18/37 | Minor | Reported | No | Table 2 |  |
| Leng 2022 | Tingling | 11/37 | 13/37 | Minor | Reported | No | Table 2 |  |
| Leng 2022 | Headache | 9/37 | 7/37 | Minor | Reported | No | Table 2 |  |
| Leng 2022 | Sleepiness | 6/37 | 5/37 | Minor | Reported | No | Table 2 |  |
| Leng 2022 | Dizziness | 0/37 | 1/37 | Minor | Reported | No | Table 2 |  |
| Leng 2022 | Mood fluctuation | 2/37 | 0/37 | Minor | Reported | No | Table 2 |  |
| Straudi 2018 | Skin redness | 13/18 | 8/17 | Minor | Reported | No | Table 4 |  |
| Straudi 2018 | Tingling | 6/18 | 8/17 | Minor | Reported | No | Table 4 |  |
| Straudi 2018 | Headache | 3/18 | 4/17 | Minor | Reported | No | Table 4 |  |
| Straudi 2018 | Sleepiness | 5/18 | 2/17 | Minor | Reported | No | Table 4 |  |
| Straudi 2018 | Trouble concentrating | 2/18 | 2/17 | Minor | Reported | No | Table 4 |  |
| Straudi 2018 | Dizziness | 1/18 | 0/17 | Minor | Reported | No | Table 4 |  |
| Straudi 2018 | Mood fluctuation | 0/18 | 1/17 | Minor | Reported | No | Table 4 |  |
| Dere 2025 | Nausea/dizziness | 3/10 | 0/9 | Minor | Reported | No | Results | Resolved after third session |
| Dere 2025 | Lower-extremity numbness | 0/10 | 1/9 | Minor | Reported | No | Results | Resolved after one day |
| Sornkaew 2024 | Tingling | NR | NR | Minor | Percent only | No | Results | 67% active and 33% sham; counts not explicitly reported |
| Sornkaew 2024 | Burning | NR | NR | Minor | Percent only | No | Results | 17% active and 0% sham |
| Sornkaew 2024 | Itching | NR | NR | Minor | Percent only | No | Results | 25% active and 20% sham |
| Jobin 2025 | Serious adverse event | 0/16 | 0/16 | Serious | Reported | No | Table 4 |  |
| Jobin 2025 | Contact dermatitis | 1/16 | 0/16 | Minor | Reported | Yes | Table 4 | Resolved after stopping treatment; participant withdrew |
| Jobin 2025 | Transient itching | 9/14 | 6/14 | Minor | Reported | No | Text / Table 5 | Severity increase greater in active group |
| Jobin 2025 | Transient tingling | NR | NR | Minor | Total only | No | Text / Table 5 | 18/28 overall; group split not reported |
| Jobin 2025 | Transient burning | NR | NR | Minor | Total only | No | Text / Table 5 | 12/28 overall; group split not reported |
| Jobin 2025 | Nausea/dizziness | NR | NR | Minor | Total only | No | Text / Table 5 | 9/28 overall; group split not reported |
| Sharma 2025 | Any adverse effect | 0/22 | 0/22 | Unspecified | Limited statement | No | Discussion | No detailed ascertainment or event table |

Notes: NR indicates that the trial did not report the relevant information. Absence of reporting was not interpreted as absence of adverse events. Six trials provided at least some safety or tolerability information. No serious adverse event was reported, but one participant in the Jobin trial withdrew because of contact dermatitis. The available sample and inconsistent ascertainment do not establish safety or exclude uncommon harms.

Abbreviations: AE, adverse event; NR, not reported; tDCS, transcranial direct current stimulation.
