# Supplementary material for: Transcranial direct current stimulation combined with rehabilitation training for chronic spinal pain: a systematic review and Meta-analysis
Source: Front Neurol. 2026 Aug 28;17:1928977. doi: 10.3389/fneur.2026.1928977 (PMC13561904; doi:10.3389/fneur.2026.1928977)
Supplement: Supplementary file 6 [file Table_6.DOCX]

**Supplementary Table S6. Detailed result-specific risk-of-bias assessments using the Cochrane Risk of Bias 2 tool**

*Estimand: effect of assignment to intervention at the immediate post-intervention assessment.*

**Table S6A. Pain intensity**

| **Study** | **Result assessed** | **D1** | **D2** | **D3** | **D4** | **D5** | **Overall** |
| --- | --- | --- | --- | --- | --- | --- | --- |
| Leng 2022 | VAS at T2 | Some concerns | Some concerns | Low risk | Some concerns | Some concerns | Some concerns |
| Lu 2024 | NRS at 4 weeks | Some concerns | Low risk | Low risk | Some concerns | Some concerns | Some concerns |
| Dere 2025 | VAS-rest at 8 weeks | Low risk | Low risk | Low risk | Some concerns | Some concerns | Some concerns |
| Qanbari 2024 | Pain intensity at post-test | Low risk | Low risk | Low risk | Low risk | Low risk | Low risk |

**Supporting rationale for the domain judgements**

| **Study** | **Result assessed** | **D1 supporting rationale** | **D2 supporting rationale** | **D3 supporting rationale** | **D4 supporting rationale** | **D5 supporting rationale/source** |
| --- | --- | --- | --- | --- | --- | --- |
| Leng 2022 | VAS at T2 | Random-number table was reported, but allocation concealment and sequence protection were not described. | A sham procedure was used, but participant/personnel blinding, adherence and trial-related deviations were insufficiently reported. | All 74 randomized participants appear in the analysis; no missing pain data were reported. | VAS is participant-reported; the article did not confirm blinding integrity, so awareness of assignment cannot be excluded. | No prospective registry or accessible analysis plan was identified; multiple outcomes and time points were available. Source: Methods 1.1–1.3 and Results tables. |
| Lu 2024 | NRS at 4 weeks | Random-number table was used; allocation concealment was not reported. Baseline characteristics were balanced. | Participants were blinded and both groups received the same exercise program; no important intervention deviations were reported. | One participant withdrew from each group (20/21 analysed per group); missingness was small and balanced. | Pain was self-reported by blinded participants, but blinding success was not tested and the trial was only single-blind. | A trial registration number was reported, but the article does not establish that the detailed outcome/analysis plan was finalized before recruitment and analysis. Source: Methods 1.1.4–2.2. |
| Dere 2025 | VAS-rest at 8 weeks | Independent assistant; computer-generated stratified block randomization; sequential opaque sealed envelopes. | Active and sham groups received the same stabilization exercises; no important deviations or differential co-interventions were reported. | All randomized participants in the active and sham arms were reported at post-treatment. | VAS was participant-reported. Sham was 0 mA and blinding effectiveness was not evaluated, so group awareness may have influenced pain ratings. | No prospective registration or accessible protocol/analysis plan was identified; many pain measures and analytic choices were available. Source: Randomization, Study Design, Outcomes and Statistical Analysis sections. |
| Qanbari 2024 | Pain intensity at post-test | Web-based block randomization was managed by an independent third party; sequentially numbered opaque sealed envelopes concealed allocation. | Participants were blinded; groups received identical sensory-motor exercise and differed only in real versus sham tDCS; intention-to-treat LMM was used. | The trial recruited 34 participants and analysed 17 per group; LMM was specified to use all available observations. | Pain was self-reported, but participants were blinded and the sham montage/procedure was matched to active stimulation. | Prospectively registered trial with prespecified primary pain outcome and published protocol/supporting files; the reported result matches the prespecified outcome. Source: Sections 2.1, 2.4, 2.6.2 and 2.7. |

**Table S6B. Disability**

| **Study** | **Result assessed** | **D1** | **D2** | **D3** | **D4** | **D5** | **Overall** |
| --- | --- | --- | --- | --- | --- | --- | --- |
| Leng 2022 | RMDQ at T2 | Some concerns | Some concerns | Low risk | Some concerns | Some concerns | Some concerns |
| Lu 2024 | ODI at 4 weeks | Some concerns | Low risk | Low risk | Some concerns | Some concerns | Some concerns |
| Dere 2025 | NBQ at 8 weeks | Low risk | Low risk | Low risk | Some concerns | Some concerns | Some concerns |
| Qanbari 2024 | Disability level at post-test | Low risk | Low risk | Low risk | Low risk | Low risk | Low risk |
| Sornkaew 2024 | MODQ at post-intervention | Low risk | Low risk | Low risk | Low risk | Low risk | Low risk |

**Supporting rationale for the domain judgements**

| **Study** | **Result assessed** | **D1 supporting rationale** | **D2 supporting rationale** | **D3 supporting rationale** | **D4 supporting rationale** | **D5 supporting rationale/source** |
| --- | --- | --- | --- | --- | --- | --- |
| Leng 2022 | RMDQ at T2 | Random-number table was reported, but allocation concealment was not described. | Sham stimulation was used, but blinding of participants/personnel and adherence/deviations were not sufficiently reported. | All 74 randomized participants appear in the analysis; no missing RMDQ data were reported. | RMDQ is participant-reported; blinding integrity was not established. | No prospective registry or prespecified analysis plan was identified; multiple outcomes/time points were available. Source: Methods 1.1–1.3. |
| Lu 2024 | ODI at 4 weeks | Random-number table was used, but allocation concealment was not described. | Participants were blinded and both groups received the same exercise treatment; no meaningful intervention deviations were reported. | One participant withdrew from each group; missingness was small and balanced. | ODI is participant-reported. Participants were blinded, but success of blinding was not examined. | Registration was reported, but prospective specification of the exact ODI analysis and reporting choice is not demonstrated in the article. Source: Methods 1.1.4–2.2. |
| Dere 2025 | NBQ at 8 weeks | Independent computer-generated block randomization with sealed opaque envelopes. | Active and sham groups received matched stabilization exercise; no differential deviations were reported. | All active and sham participants were reported at post-treatment. | NBQ is participant-reported and includes pain and psychosocial items. Sham was 0 mA and blinding effectiveness was not tested. | No prospective registry/protocol was identified; several functional and symptom measures and post-hoc analyses were available. Source: Randomization, Outcomes and Statistical Analysis. |
| Qanbari 2024 | Disability level at post-test | Independent web randomization, block size 4, and sequential opaque sealed envelopes. | Participants were blinded; both groups received identical sensory-motor training; ITT LMM was used. | Seventeen participants per group were analysed; LMM accommodated any incomplete observations. | The disability scale was participant-reported, but participant blinding and matched sham reduce concern. | Disability was a prespecified primary outcome in a prospectively registered trial with protocol files. Source: Sections 2.1, 2.4 and 2.7. |
| Sornkaew 2024 | MODQ at post-intervention | An independent researcher randomized participants using sealed envelopes after baseline testing; participants and other researchers were unaware of assignment. | Active and sham groups received the same motor-control exercise; sham fade-in/out was designed to maintain blinding; no deviations were reported. | All 22 participants completed the 6-week intervention. | MODQ is participant-reported, but participants and researchers were blinded. | The trial was prospectively registered and disability was explicitly identified as a secondary outcome; the reported result corresponds to that outcome. Source: Participants/randomization, Clinical Outcomes and Study Registration sections. |

**Table S6C. Quality of life**

| **Study** | **Result assessed** | **D1** | **D2** | **D3** | **D4** | **D5** | **Overall** |
| --- | --- | --- | --- | --- | --- | --- | --- |
| Leng 2022 | EQ-5D at T2 | Some concerns | Some concerns | Low risk | Some concerns | Some concerns | Some concerns |
| Straudi 2018 | EQ-5D change at T2 | Low risk | Low risk | Some concerns | Low risk | Low risk | Some concerns |
| Sornkaew 2024 | SF-36 at post-intervention | Low risk | Low risk | Low risk | Low risk | Low risk | Low risk |
| Jobin 2025 | EQ-5D group×time result | Low risk | Low risk | Some concerns | Low risk | Low risk | Some concerns |

**Supporting rationale for the domain judgements**

| **Study** | **Result assessed** | **D1 supporting rationale** | **D2 supporting rationale** | **D3 supporting rationale** | **D4 supporting rationale** | **D5 supporting rationale/source** |
| --- | --- | --- | --- | --- | --- | --- |
| Leng 2022 | EQ-5D at T2 | Random-number table reported; concealment not described. | Sham used, but blinding/adherence/deviations insufficiently reported. | No missing EQ-5D data reported. | Participant-reported QoL; blinding integrity unclear. | No registry/protocol and several outcomes/time points. Source: Methods and outcome tables. |
| Straudi 2018 | EQ-5D change at T2 | Block randomization generated externally; allocation list managed outside the research group. | Participants, investigators, therapists and physicians were blinded; matched sham was used. | One participant discontinued and missing values were imputed using last observation carried forward, which may bias a small pilot trial. | Validated self-reported EQ-5D completed under double blinding. | Prospectively registered trial; QoL was prespecified and reported at stated time points. Source: Methods, Data Analysis and CONSORT flow. |
| Sornkaew 2024 | SF-36 at post-intervention | Independent randomization with sealed envelopes after baseline; participants and researchers blinded. | Matched sham and identical motor-control exercise; no deviations reported. | All 22 participants completed follow-up. | Participant-reported SF-36 under blinded allocation. | Prospectively registered; SF-36 specified as a secondary outcome and reported. Source: Participants, Clinical Outcomes and Registration. |
| Jobin 2025 | EQ-5D group×time result | Sequentially numbered sealed envelopes in random blocks; allocation held by an uninvolved researcher. | Participants, assessors and care providers were blinded; only the tDCS administrator was unblinded. Blinding efficacy was assessed. | Four participants discontinued; mixed-effects models used available data. Attrition was balanced at treatment completion but remains relevant in a small pilot study. | Validated EQ-5D under effective blinding; only 39% correctly guessed assignment with no between-group difference. | Prospectively registered and protocol published; QoL analysis used prespecified mixed-effects methods. Source: Design, Randomization, Statistical Analysis and Results. |

Domain definitions: D1, bias arising from the randomization process; D2, bias due to deviations from intended interventions; D3, bias due to missing outcome data; D4, bias in measurement of the outcome; D5, bias in selection of the reported result. Judgements are result-specific rather than global study-level ratings.

Judgement categories: Low risk; Some concerns; High risk. No high-risk result contributed to either primary meta-analysis.
